# (1,3)-β-d-Glucan-based empirical antifungal interruption in suspected invasive candidiasis: a randomized trial

**DOI:** 10.1186/s13054-020-03265-y

**Published:** 2020-09-05

**Authors:** Gennaro De Pascale, Brunella Posteraro, Sonia D’Arrigo, Giorgia Spinazzola, Rita Gaspari, Giuseppe Bello, Luca Maria Montini, Salvatore Lucio Cutuli, Domenico Luca Grieco, Valentina Di Gravio, Giulia De Angelis, Riccardo Torelli, Elena De Carolis, Mario Tumbarello, Maurizio Sanguinetti, Massimo Antonelli

**Affiliations:** 1 Dipartimento di Scienza dell’Emergenza, Anestesiologiche e della Rianimazione – UOC di Anestesia, Rianimazione, Terapia Intensiva e Tossicologia Clinica, Fondazione Policlinico Universitario A. Gemelli IRCCS, Largo A. Gemelli 8, 00168 Rome, Italy; 2 Università Cattolica del Sacro Cuore, Istituto di Anestesia e Rianimazione, Largo F. Vito 1, 00168 Rome, Italy; 3 Dipartimento di Scienze Gastroenterologiche, Endocrino-Metaboliche e Nefro-Urologiche, Fondazione Policlinico Universitario A. Gemelli IRCCS, Largo A. Gemelli 8, 00168 Rome, Italy; 4 Università Cattolica del Sacro Cuore, Istituto di Patologia Medica e Semeiotica Medica, Largo F. Vito 1, 00168 Rome, Italy; 5 Università Cattolica del Sacro Cuore, Istituto di Microbiologia, Largo F. Vito 1, 00168 Rome, Italy; 6 Dipartimento di Scienze di Laboratorio e Infettivologiche, UOC di Microbiologia, Fondazione Policlinico Universitario A. Gemelli IRCCS, Largo A. Gemelli 8, 00168 Rome, Italy; 7 Dipartimento di Scienze di Laboratorio e Infettivologiche , UOC di Malattie Infettive, Fondazione Policlinico Universitario A. Gemelli IRCCS, Largo A. Gemelli 8, 00168 Rome, Italy; 8 Università Cattolica del Sacro Cuore, Istituto di Malattie Infettive, Largo F. Vito 1, 00168 Rome, Italy

**Keywords:** Sepsis, *Candida* infection, Biomarker, (1,3)-β-d-Glucan, Antifungal therapy

## Abstract

**Background:**

(1,3)-β-d-Glucan has been widely used in clinical practice for the diagnosis of invasive *Candida* infections. However, such serum biomarker showed potential to guide antimicrobial therapy in order to reduce the duration of empirical antifungal treatment in critically ill septic patients with suspected invasive candidiasis.

**Methods:**

This was a single-centre, randomized, open-label clinical trial in which critically ill patients were enrolled during the admission to the intensive care unit (ICU). All septic patients who presented invasive *Candida* infection risk factors and for whom an empirical antifungal therapy was commenced were randomly assigned (1:1) in those stopping antifungal therapy if (1,3)-β-d-glucan was negative ((1,3)-β-d-glucan group) or those continuing the antifungal therapy based on clinical rules (control group). Serum 1,3-β-d-glucan was measured at the enrolment and every 48/72 h over 14 days afterwards. The primary endpoint was the duration of antifungal treatment in the first 30 days after enrolment.

**Results:**

We randomized 108 patients into the (1,3)-β-d-glucan (*n* = 53) and control (*n* = 55) groups. Median [IQR] duration of antifungal treatment was 2 days [1–3] in the (1,3)-β-d-glucan group vs. 10 days [6–13] in the control group (between-group absolute difference in means, 6.29 days [95% CI 3.94–8.65], *p* < 0.001). Thirty-day mortality was similar (28.3% [(1,3)-β-d-glucan group] vs. 27.3% [control group], *p* = 0.92) as well as the overall rate of documented candidiasis (11.3% [(1,3)-β-d-glucan group] vs. 12.7% [control group], *p* = 0.94), the length of mechanical ventilation (*p* = 0.97) and ICU stay (*p* = 0.23).

**Conclusions:**

In critically ill septic patients admitted to the ICU at risk of invasive candidiasis, a (1,3)-β-d-glucan-guided strategy could reduce the duration of empirical antifungal therapy. However, the safety of this algorithm needs to be confirmed in future, multicentre clinical trial with a larger population.

**Trial registration:**

ClinicalTrials.gov, NCT03117439, retrospectively registered on 18 April 2017

## Backgrounds

Intensive care unit (ICU) physicians usually prescribe empirical antifungals relying upon clinical risk factors [1] or prediction rules (i.e. a *Candida* score [CS] of ≥ 3 and a *Candida* colonization index [CCI] of ≥ 0.5), which, unfortunately, do not always address a population with high rates of invasive candidiasis [2–5].

Although supported by a low-quality evidence, current guidelines recommend to stop antifungals after 5 to 10 days of therapy, according to final microbiological diagnosis, patients’ clinical conditions and the results of non-culture-based diagnostic assays [6, 7]. Nonetheless, the unrestrictive use of antifungals, without evidence of confirmed infection, is a driver of increased costs and drug-related side effects, potentially influencing *Candida* ecology and overall antifungal susceptibility. In a seminal 1-day multicentre observational study [8], 7.5% ICU patients underwent systemic antifungals: two third of them had no documented infection and no survival benefit was observed in such population. These results have been recently confirmed in a large randomized trial where empirical therapy with micafungin in patients with ICU-acquired sepsis, *Candida* colonization and multiple organ failure did not increase the fungal infection-free survival at day 28 [9]. Indeed, an early (≤ 5 days) empirical antifungal de-escalation strategy in patients with suspected invasive *Candida* infection (ICI) is increasingly adopted and recent observational data support the safety of such approach in critically ill patients [10].

Among non-culture-based diagnostic tests, (1,3)-β-d-glucan (BDG) has been used as a biomarker for prompt ICI diagnosis and as a guide to discontinue empirical antifungal therapy [11–15]. Interestingly, in a recent pivotal, randomized study, the BDG assay, used in combination with mannan/anti-mannan serum assays, significantly increased (54% vs. 2%, *p* < 0.001) the rate of early antifungal therapy interruption without influencing ICI-free 28-day survival [16].

Since 2011, we have been implementing the use of BDG not only to hasten the time for the diagnosis and treatment of potential ICIs but also as a tool for antifungal stewardship [17], reporting the possibility to avoiding or shortening duration of the empirical antifungal treatment in more than 70% of potentially treatable cases [18].

The aim of the present study was to test whether the BDG used as a decision-making tool for empirical antifungal therapy management may be effective in reducing the duration of antifungal treatments in critically ill septic patients with suspected ICIs.

## Methods

### Study design and patients

This open-label, double-arm, randomized controlled trial was conducted in the 18-bed general ICU and in the 13-bed surgical ICU of a 1500-bed tertiary teaching hospital in Rome, from July 1, 2016, to June 30, 2018. According to Italian law, the protocol was approved by the Università Cattolica del Sacro Cuore Ethics Committee (approval number UCSC20980/16), and written informed consent was obtained from the patient or the legally authorized representative. The trial was registered on www.clinicaltrials.gov (NCT03117439). The manuscript was prepared according to the CONSORT statement.

ICU patients older than 18 years were assessed for eligibility if they developed sepsis according to the Sepsis-3 definitions [19, 20] while ongoing broad-spectrum antibiotic therapy (i.e. receiving at least two antibiotics or one active against MDR pathogens in patients with sepsis persisting from more than 48 h). Patients should also match the following conditions: (1) ICU stay ≥ 48 h with an expected length of stay of 48 h, (2) mechanical ventilation (MV), (3) presence of central venous catheter (CVC)/arterial line (AL), (4) presence of septic shock and (5) CS ≥ 3 or CCI ≥ 0.5 if septic shock was absent (eFig. 1). Patients were excluded if they (1) had a diagnosis of complicated ICI, (2) were already ongoing any type of antifungal therapy, (3) were immunocompromised (long-term immunosuppressive or steroid therapy; acquired immunodeficiency syndrome [AIDS]; white blood cell count (WBC) < 1000/mmc or neutrophils < 500/mmc), (4) were pregnant, (5) were already enrolled in other interventional studies, (6) if BDG serum test was not available (for the BG group) and (7) in absence of informed consent. Patients who died in the first 24 h were also excluded.

### Randomization and masking

In each ICU, the local investigators enrolled participants in the study. Patients were randomized in a 1:1 ratio to treatment groups according to a computer-generated random assignment. Allocation was issued using opaque, sealed, numbered envelopes. No masking was used after randomization. All the investigators were involved in the study planning, protocol design, trial running and final report writing.

### Procedures

All patients received a first dose of antifungal therapy and had serum (1,3)-β-d-glucan levels measured at baseline. In the patients assigned to the BDG group, antifungal therapy was stopped in the presence of a first negative (1,3)-β-d-glucan value (< 80 pg/mL) and maintained in case of a positive result. BDG serum levels were checked every 48/72 h during the first 14 days after enrolment, stopping ongoing antifungals after a negative result and restarting them in case of a result ≥ 80 pg/mL. In the patients assigned to the control group, the decision regarding the duration of antifungal therapy was left to the attending physician, according to current guidelines for empirical antifungal therapy [11, 12]. In the control group, BDG levels were not measured after the baseline and their use was not allowed for antifungal stewardship. All documented ICIs were treated according to current guidelines [6, 7]. All data were recorded on the electronic ICU charts (Digistat®) and computerized microbiology laboratory databases (see Additional file 1 for further details).

### Outcome measures

The primary outcome was the duration of antifungal therapy (days) during the first 30 days after study inclusion. Secondary endpoints were 30-day mortality, ICU mortality, hospital mortality, antifungal stopping rate, duration of MV, duration of ICU length of stay, prevalence of ICIs and their outcome and cost analysis including antifungal types and BDG test performed.

### Microbiological analysis

Serum recovered from the patients’ blood samples was tested for BDG (Fungitell; Associates of Cape Cod Inc., Falmouth, MA, USA) according to the manufacturer’s instructions. All BDG tests had a turnaround time ≤ 24 h, with the exception of samples from the patients enrolled on Saturday and Sunday. The concentration of BDG in each sample was automatically calculated, and 80 pg/mL was used as a positive cut-off, according to the manufacturer (Fungitell) indications (see additional file 1 for further details) [17].

### Statistical analysis

**Fig. 1:**
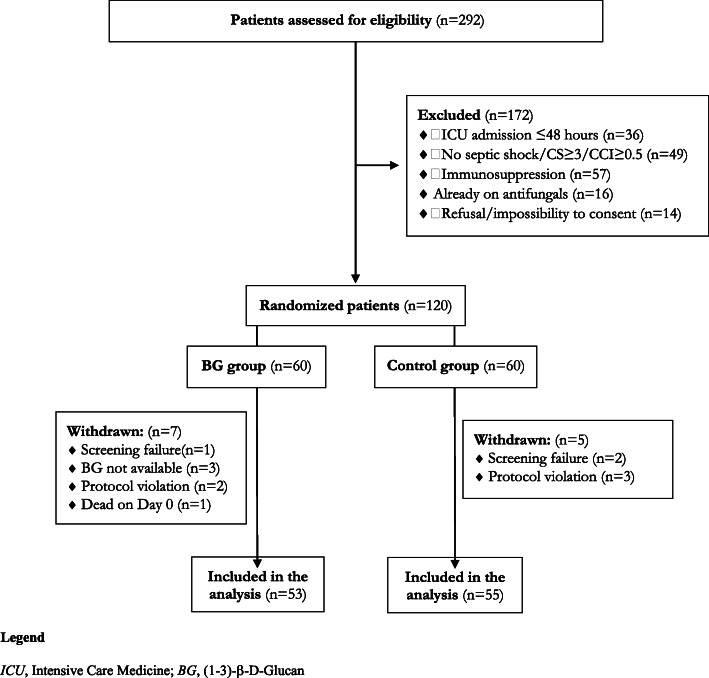
Flow chart of the study inclusion process

This aimed to test whether the BDG-guided strategy was superior in terms of antifungal duration, as assessed by the number of days with antifungals during the first 30 days from enrolment. According to previously published observational data [18], the study was designed to enrol at least 96 patients to obtain, with a power of 95%, a 30% (3 days) difference in duration of antifungal therapy between the two groups. We assumed a standard deviation (SD) of 4 days in both groups and an α error of 0.05 and an estimated baseline duration of 10 days. To account for an approximate 20% dropout rate, we set a total number of 120 patients (Fig. 1). All statistical analyses were performed using MedCalc software, version 12.2.1 (MedCalc®, MariaKerke, Belgium). Graphing of data was undertaken using Prism version 6.0 for Windows (graphPad Software, San Diego, CA) (see Additional file 1 for further details).

## Results

### Study patients

Two hundred ninety-two patients with sepsis and risk factors for ICI were assessed for eligibility, of whom 120 were enrolled and randomly assigned to the BDG group (*n* = 60) and the control group (*n* = 60). Seven patients in the BDG group and five in the control group were subsequently excluded from the analysis (Fig. 1).

The two groups had similar characteristics at baseline (Table 1) although patients in the control group were more frequently admitted after surgery and had less circulatory failure (45.5% vs. 26.4%, *p* = 0.05 and 9.1% vs. 24.5%, *p* = 0.04, respectively). Fifty-five out of 108 patients presented a CS value ≥ 3, with abdominal surgery and multifocal *Candida* colonization as leading risk factors (*n* = 70, 64.8% and *n* = 39, 36.1%, respectively). No more than one third of the enrolled patients had a CCI ≥ 0.5, and *Candida albicans* was the most prevalent isolated species (22 out of 35 isolates, 62.9%). Sixty patients (55.6%) received echinocandins as first-line antifungal agents, without significant differences in terms of either drug type or allocation group. In 20 patients, a bacterial bloodstream infection was detected, mainly due to Gram-negative bacteria (*n* = 16) (Table 1).

**Table 1:** Baseline patient characteristics

Variable	BDG group (*n* = 53)	Control group (*n* = 55)	*p* value
Demographics and comorbidities			
Age, years	62 [47–75]	68 [52.5–73]	0.49
Male sex, *N* (%)	34 (64.2)	32 (58.2)	0.56
Medical admission, *N* (%)	31 (58.5)	26 (47.3)	0.26
-Respiratory failure, *N* (%)	13 (24.5)	17 (30.9)	0.52
-**Circulatory failure,** ***N*** **(%)**	**13 (24.5)**	**5 (9.1)**	**0.04**
-Other organ failure, *N* (%)*	5 (9.4)	4 (7.3)	0.96
**Surgical admission,** ***N*** **(%)**	**14 (26.4)**	**25 (45.5)**	**0.05**
Trauma admission, *N* (%)	8 (15.1)	4 (7.3)	0.23
SAPS II score	43 [33–57]	42 [32.25–52.75]	0.59
Charlson score	3 [1–6]	4 [1–6.75]	0.57
-Cardiovascular diseases, *N* (%)	11 (20.8)	7 (12.7)	0.31
-COPD, *N* (%)	8 (15.1)	10 (18.2)	0.8
-Chronic renal failure, *N* (%)	8 (15.1)	7 (12.7)	0.78
-Diabetes, *N* (%)	7 (13.2)	14 (25.5)	0.15
-Chronic liver failure, *N* (%)	4 (7.5)	1 (1.8)	0.2
**Presenting features and risk factors for ICI at randomization**			
Hospital LOS before randomization (days)	9 [3.75–15.25]	8 [4–13.75]	0.79
ICU LOS before randomization (days)	3 [2–10]	3 [2–5]	0.4
MV duration before randomization (days)	3 [1–8.25]	2 [1–4.75]	0.67
Vasopressors duration before randomization (days)	0 [0–3]	0 [0–3]	1
SOFA score	7 [4.75–11.25]	7 [4–10]	0.19
Septic shock, *N* (%)	27 (50.9)	26 (47.3)	0.85
AKI requiring CRRT, *N* (%)	15 (28.3)	7 (12.7)	0.06
*Candida* score ≥ 3, *N* (%)	26 (49.1)	29 (52.7)	0.85
-Abdominal surgery, *N* (%)	30 (56.6)	40 (72.7)	0.11
-Multifocal *Candida* colonization, *N* (%)	21 (39.6)	18 (32.7)	0.55
-Total parenteral nutrition, *N* (%)	5 (9.4)	18 (32.7)	**0.004**
*Candida* colonization index ≥ 0.5, *N* (%)	17 (32.1)	18 (32.7)	1
-*Candida albicans*, *N* (%)	12 (22.6)	10 (18.2)	0.74
-Non-*C. albicans Candida* species, *N* (%)**	5 (9.4)	8 (14.5)	0.6
Invasive *Candida* infection, *N* (%)^#^	6 (11.3)	5 (9.1)	0.95
Bacterial bloodstream infection, *N* (%)^##^	11 (20.8)	9 (16.4)	0.73
-Gram-positive bacteria, *N* (%)	3 (5.7)	1 (1.8)	0.57
-Gram-negative bacteria, *N* (%)	8 (15.1)	8 (14.5)	0.85
Echinocandins as initial antifungals, *N* (%)	33 (62.3)	27 (49.1)	0.24
-Caspofungin, *N* (%)	16 (30.2)	16 (29.1)	0.93
-Anidulafungin, *N* (%)	17 (32.1)	11 (20)	0.23
Fluconazole as initial antifungal, *N* (%)	20 (37.7)	28 (50.9)	0.24

Data are presented as median [IQR], unless otherwise indicated

*BDG* (1–3)-β-d-glucan, *SAPS II* Simplified Acute Physiology Score, *COPD* chronic obstructive pulmonary disease, *ICI* invasive *Candida* infection, *LOS* length of stay, *ICU* intensive care unit, *MV* mechanical ventilation, *SOFA* Sequential Organ Failure Assessment, *AKI* acute kidney injury, *CRRT* continuous renal replacement therapy, *CVC* central venous catheter, *IQR* interquartile range

*Neurological failure (*n* = 6), liver failure (*n* = 2), renal failure (*n* = 1)

**Candida glabrata (*n* = 4), *Candida tropicalis* (*n* = 3), *Candida dubliniensis* (*n* = 3), *Candida parapsilosis* (*n* = 3)

^#^See eTable 4 for further details

^##^Gram-positive bacteria (*Enterococcus* spp., *n* = 3; *Staphylococcus aureus*, *n* = 1); Gram-negative bacteria (*Escherichia coli*, *n* = 5; *Klebsiella pneumoniae*, *n* = 4; *Enterobacter* spp., *n* = 3; *Acinetobacter Baumannii*, *n* = 2; *Proteus mirabilis*, *n* = 1; *Bacteroides fragilis*, *n* = 1)

### Primary and secondary endpoints

**Fig. 2:**
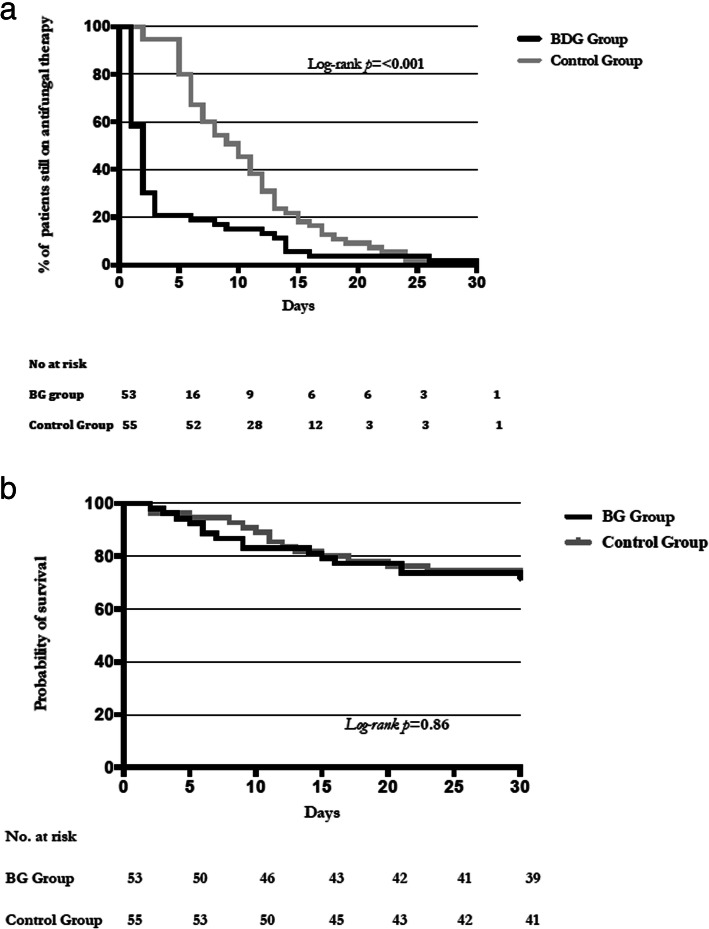
**a** Kaplan-Meier plots showing the evolution with time of the percentage of patients who remained on antifungals in the (1,3)-β-d-glucan and control groups. **b** Probability of survival from study inclusion (day 0) through day 30 for patients in the (1,3)-β-d-glucan and control groups

The duration of antifungal therapy within the first 30 days after enrolment was significantly lower in the BDG group (median [interquartile range, IQR] 2 [1–3] days), compared with the control group (10 [6–13] days) (between-group absolute difference in means 6.29 days, 95% CI [3.94 to 8.65], *p* < 0.001) (Table 2). The antifungal stopping rate was significantly higher in the BDG group compared with the control group at day 5 (*N* = 37, 69.8% vs. *N* = 3, 5.5%, respectively), at day 10 (*N* = 44, 83% vs. *N* = 27, 49.1%, respectively) and at day 15 (*N* = 47, 88.7% vs. 43, *N* = 78.2%, respectively) (HR 2.06, 95% CI 1.83–4.12, *p* < 0.001 by log-rank test) (Fig. 2a). The Kaplan-Meier survival probability at day 30 did not differ between the two groups (HR 1.07, 95% CI 0.52–2.18, *p* = 0.86 by log-rank test), as well as the rate of ICU and hospital mortality (30.2% vs. 30.9%, *p* = 0.89 and 35.9% vs. 32.7%, *p* = 0.88, respectively) (Fig. 2b). This was confirmed by Cox regression analysis, evaluating the role of potential confounders (type of admission, Charlson score, SAPS II score, SOFA score, occurrence of septic shock) (HR 0.91, 95% CI [0.43–1.91], *p* = 0.8). Other secondary outcomes did not differ between the two groups (Table 2) (see Additional file 1 for further details).

**Table 2:** Outcome measures in the (1–3)-β-d-glucan (BDG) and the control groups

Variable	BDG group (*n* = 53)	Control group (*n* = 55)	Between-group absolute difference in means (95% CI)	*p* value
**Primary outcome**				
Duration of antifungal therapy, days	2 [1–3]	10 [6–13]	6.29 (3.94 to 8.65)	**< 0.001**
**Secondary outcomes**				
30-day mortality, *N* (%)	15 (28.3)	15 (27.3)	− 1% (− 16.89 to 18.93)	0.92
ICU mortality, *N* (%)	16 (30.2)	17 (30.9)	0.7% (− 17.7 to 18.97)	0.89
Hospital mortality, *N* (%)	19 (35.9)	18 (32.7)	− 3.2% (− 15.7 to 21.93)	0.88
Subsequent ICI, *N* (%)*	0	2 (3.6)	3.6% (− 3.83 to 12.47)	0.5
Hospital LOS, days	35 [23.75–55.25]	38 [20–59.5]	− 7.41 (− 21.55 to 6.73)	0.87
ICU LOS, days	18 [7.75–24.25]	13 [7–26]	− 0.5 (− 6.95 to 5.95)	0.23
Mechanical ventilation duration, days	9 [4.75–17.25]	9 [3.25–19.75]	3.21 (− 2.05 to 8.46)	0.97
Vasopressors duration, days	4 [0.75–8.25]	3 [0–11]	0.06 (− 2.95 to 3.07)	0.6
Total antifungals costs, €	110 [2.64–708]	113.2 [9.68–1255.6]	318.63 (− 310.1 to 947.3)	0.24
Echinocandins cost, €	708 [185.6–1071.5]	1320 [618.5–30,149.5]	937.05 (− 64.2 to 1938.3)	0.07
BG cost, € mean ± SD	80.8 ± 20.4	–	–	–

Data are presented as median (IQR) and *N* (%). Between-group absolute differences are calculated using the mean values, percentage differences and 95% CIs

*BDG* (1–3)-β-d-glucan, *ICU* intensive care unit, *ICI* invasive *Candida* infection, *LOS* length of stay, *€* euro, *IQR* interquartile range

*See eTable 1 for further details

### Invasive *Candida* infections and (1,3)-β-d-glucan results

**Fig. 3:**
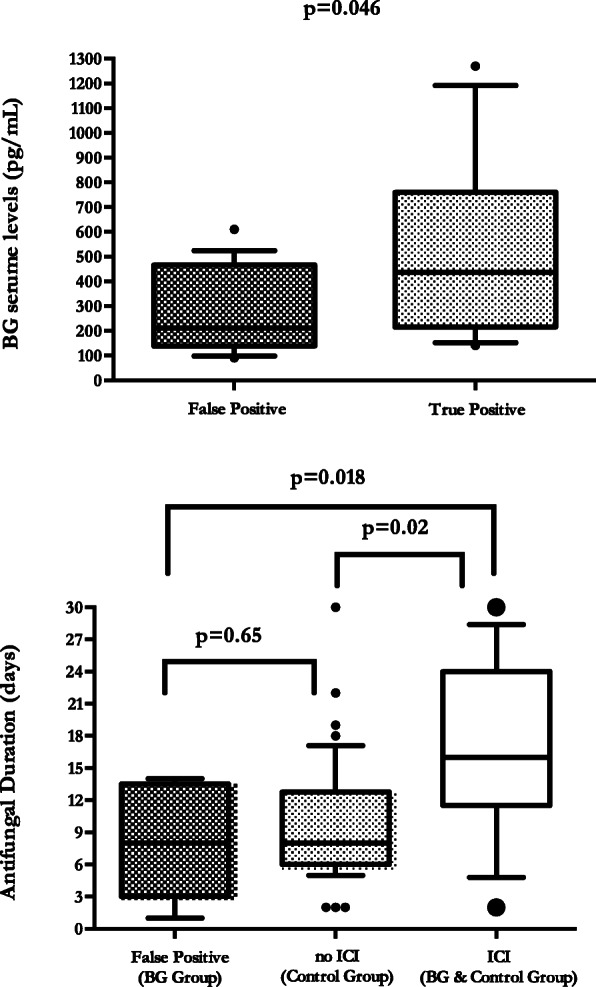
**a** (1,3)-β-d-Glucan results for patients with and without invasive *Candida* infections. **b** Duration of antifungal therapy among intensive care unit patients according to the absence or presence of invasive *Candida* infections and the positivity of the (1,3)-β-d-glucan assay

No false negative BDG results were obtained, and thirteen ICIs were diagnosed during the study period: 11 at enrolment (six in the BDG group and five in the control group) and 2 during the follow-up period (both in the control group). Eleven patients were candidaemic and two had intra-abdominal candidiasis without positive blood cultures. Overall mortality was 46.2% without differences between the two groups in terms of type of infection, clinical severity and main outcomes (eTable 1). Baseline BDG levels were detected in all but three patients in the control group. Nineteen subjects had BDG values ≥ 80 pg/mL in the absence of ICI (i.e. false positive), but their levels were lower compared with true BG-positive sera (292.9 ± 173.4 pg/mL vs. 492 ± 350.5 pg/mL, *p* = 0.046) (Fig. 3a). Additionally, excluding negative results, BDG initial levels were relatively high, especially in the BDG group (eFigure 3). The duration of antifungal therapy in patients randomized to the BDG group with false positive results did not differ from that of patients in the control group without ICI (8 [3–13.25] days vs. 8 [6–12.5] days, *p* = 0.65) and was significantly lower compared with that of patients with ICI (8 [3–13.25] days vs. 16 [11.75–24] days, *p* = 0.018) (Fig. 3b).

## Discussion

In this randomized controlled open-label study, the use of BDG as an antifungal stewardship tool in critically ill septic patients at risk of ICI did significantly reduce the duration of antifungal therapy without adverse clinical outcome. BDG diagnostic performance was higher compared with commonly used clinical and microbiological algorithms, and BDG serum levels of patients with false positive results were significantly lower than those observed in patients with ICI.

During the last years, (1,3)-β-d-glucan clinical use has been implemented as a non-invasive diagnostic test for invasive candidiasis in both non-neutropenic and onco-haematological patients [21]. In a cohort of septic critically patients, the use of the BDG allowed an early diagnosis of ICI with a negative predictive value (NPV) of nearly 99%, hastening the diagnosis of candidaemia of 24–72 h compared with standard microbiological results [17]. Moreover, in 166 septic patients (73 with candidaemia and 93 with bacteraemia), the combination of procalcitonin (< 2 ng/mL) with BDG (≥ 80 pg/mL) has been observed to increase the overall positive predictive value (PPV) to 96% and the NPV to 95% [22].

Our results show that in high-risk critically ill septic patients, the antifungal empirical therapy may be shortened when at least one BDG is negative, which would rule out the presence of an invasive candidiasis. Although the study population included patients at high risk of ICI, mainly involving surgical patients, these data are in line with recent studies investigating the reliability of such test as a decision-making tool for empirical antifungal interruption in patients at risk of ICI [23]. In two observational investigations, such approach allowed safe empirical antifungal discontinuation as confirmed by the definite negative findings from blood and other sterile site cultures [13, 18], thus shortening empirical antifungal therapy exposition in about 90% of patients with negative BDG [18]. Similarly, the combination of BDG with *Candida albicans* germ tube antibody (CAGTA) showed a 100% sensitivity in a population of septic patients (mainly underwent gastrointestinal surgery), allowing antifungal discontinuation in one third of these cases [24].

Only one randomized trial had investigated the feasibility of a biomarker-based strategy for early interruption of antifungal treatment in high-risk critically ill patients [16]. In that paper, 109 ICU patients with risk factors for ICI were randomized to a strategy where the duration of antifungals was decided based on BDG, mannan and anti-mannan assays or to a standard 14-day empirical treatment. Interestingly, empirical antifungal therapy was stopped earlier in the biomarker group compared to the control group (54% vs. 2%, *p* < 0.001), with lower duration in the former (6 vs. 14 days, *p* < 0.001, respectively) and no impact on ICI rate, MV-free days, ICU length of stay and mortality. Although in line with the above results, our study provides new information on the clinical feasibility of a biomarker-based antifungal stewardship and addresses some different features. First, our study was more focussed on septic patients at risk for ICI, while all the other study referred to general critically ill patients. Second, we used a single biomarker (i.e. BDG-based) strategy where the BDG assay was performed the day of enrolment and at least every 48/72 h during the first 14 days. In patients randomized to the BDG group, with the exception of those enrolled on Saturday and Sunday, the result was available in the next 24 h and, in the presence of a negative result, antifungal therapy was promptly stopped. This strategy explains the really short duration of therapy in patients managed with the BDG algorithm (median [IQR] 2 days [1–3]), where in most cases only the first antifungal loading dose was administered. However, we observed an unbalanced distribution of surgical patients (mainly among controls), leading to a potential bias in the de-escalation approach. Nevertheless, compared with other investigations, also patients in the control group received a shorter course of empirical therapy [25, 26]. Given the detrimental clinical impact of inadequate empirical antifungal treatment in critically ill patients with ICI, current guidelines address the importance of a prompt and adequate empirical antifungal treatment in all high-risk patients, but clinical and microbiological indications for antifungal de-escalation in patients without evidence of infection are still a matter of debate [6, 27]. The most recent IDSA guidelines [12] recommend a minimal duration of empirical antifungal therapy of 4–5 days, depending on clinical response and microbiological results including non-culture-based diagnostic tests with a high NPV. Interestingly, in a recent observational study, antifungal de-escalation was performed in 20% of 190 patients, significantly shortening treatment duration (median [IQR] 6 [5–18] vs. 13 [7–15], *p* = 0.02), without any adverse clinical outcome [10]. Similarly, two recent large-scale studies failed to demonstrate outcome benefits for empirical systemic antifungal therapy in non-immunocompromised critically ill patients [9, 25]. In our study, the decision to discontinue the antifungal therapy was not protocolized and was left to the attending physician, based on patients’ clinical conditions and final microbiological diagnosis, with the indication to keep the 5–14-day interval (time to definite microbiological results and recommended duration of uncomplicated episodes, respectively). This approach was previously applied in another observational study from our group where the duration of antifungal empirical therapy for patients with risk factors but without evidence of ICI ranged from 4 and 11 days [18].

One of the drawbacks of BDG use in such clinical setting is the possibility of false positive results. These may be associated with different clinical conditions, including renal replacement therapy (RRT), packing with surgical gauzes, concomitant administration of albumin, immunoglobulins, beta-lactam antibiotics or Gram-positive bloodstream infections [28, 29]. False positive results usually show lower serum levels compared with true positive assays and, although in the absence of a clear clinical threshold, they are not influenced by the administration of antifungals [22]. In our study, the rate of false positive BDG results was 18.3% and, with the exception of Ig administration, it was not associated with other usual confounding factors. In addition, all the disposable material used for test analysis was BDG free. However, this feature did not represent a clinical issue, since the duration of antifungal therapy in such patients was actually the same of controls (8.2 ± 1.7 days vs. 9.6 ± 0.8 days, *p* = 0.65). Additionally, BDG initial levels were high in both groups (higher in the BDG group), and this aspect may be explained by the selection bias deriving from the exclusion of all negative results. Our data are in contrast with the EMPIRICUS randomized clinical trial, where BDG levels were not significantly different between patients with candidaemia vs. those with multiple colonization [9, 17, 18]. The observed differences may be explained by the availability, in our study, of a single and dedicated microbiological laboratory for BDG assay testing, thus minimizing the possibility of environmental glucan contaminations. Finally, our study supports the use of a BDG-based antifungal stewardship strategy to reduce antifungal exposure, limiting its effect on ecological pressure (emergence of resistant *C. albicans* strains and non-*C. albicans Candida* species), drug toxicity, adverse drug interactions and echinocandin-driven costs [30, 31].

This study has a number of limitations. First, the trial was unblinded and monocentric, so the results, especially secondary outcomes, may not be generalized to other settings with different epidemiological patterns and laboratory resources. Second, the overall rate of invasive candidiasis was not high, and this may be explained by the specific case-mix of the study where only part of the patients belonged to the categories at highest risk of ICI as recurrent gastrointestinal leakage and acute necrotizing pancreatitis. In addition, surgical admissions were more frequent among controls, leading to a potential bias regarding the observed de-escalation rate. Third, there was not a fixed timepoint for BDG assessment but a window of at least 24 h. Further, therapy duration in the control group was not standardized and mainly represented by surgical cases although reflecting a common clinical approach in the daily real life. Finally, the sample size was not appropriate to definitely address the safety of such strategy.

## Conclusions

In conclusion, this is the first randomized trial where an algorithm based only on BDG results was evaluated as antifungal stewardship tool in severe patients with sepsis. This study shows that a reduction in the antifungal therapy duration and consumption in critically ill patients at risk of ICI (especially surgical ones) can be obtained by the use of this BDG-guided algorithm. A larger multicentre randomized study is warranted to confirm the efficacy and safety of such approach in the ICU setting.

## Supplementary information

**Additional file 1.** Including further details on Methods, Results and six tables (eTable 1, eTable 2, eTable 3, eTable4, eTable 5, eTable 6) and three figures (eFigure 1, eFigure 2, eFigure 3).

## Data Availability

The datasets used and/or analyzed during the current study are available from the corresponding author on reasonable request.

## Abbreviations

ICU: Intensive care unit
CS: *Candida* score
CCI: *Candida* colonization index
ICI: Invasive *Candida* infection
BDG: (1,3)-β-d-Glucan
MV: Mechanical ventilation
CVC: Central venous catheter
AL: Arterial line
AIDS: Acquired immunodeficiency syndrome
WBC: White blood cell count
SD: Standard deviation
IQR: Interquartile range
NPV: Negative predictive value
PPV: Positive predictive value
CAGTA: *Candida albicans* germ tube antibody

## References

[1] Ostrosky-Zeichner L, Shoham S, Vazquez J, Reboli A, Betts R, Barron M, Schuster M, Judson M, Revankar S, Caeiro J (2014). MSG-01: a randomized, double-blind, placebo-controlled trial of caspofungin prophylaxis followed by preemptive therapy for invasive candidiasis in high-risk adults in the critical care setting. Clin Infect Dis.

[2] León C, Ruiz-Santana S, Saavedra P, Almirante B, Nolla-Salas J, Alvarez-Lerma F, Garnacho-Montero J, León M, EPCAN Study Group (2006). A bedside scoring system (“Candida score”) for early antifungal treatment in nonneutropenic critically ill patients with Candida colonization. Crit Care Med.

[3] León C, Ruiz-Santana S, Saavedra P, Galván B, Blanco A, Castro C, Balasini C, Utande-Vázquez A, González de Molina F, Blasco-Navalproto M (2009). Usefulness of the “Candida score” for discriminating between Candida colonization and invasive candidiasis in non-neutropenic critically ill patients: a prospective multicenter study. Crit Care Med.

[4] Pittet D, Monod M, Suter P, Frenk E, Auckenthaler R (1994). Candida colonization and subsequent infections in critically ill surgical patients. Ann Surg.

[5] Eggimann P, Pittet D (2014). Candida colonization index and subsequent infection in critically ill surgical patients: 20 years later. Intensive Care Med.

[6] Martin-Loeches I, Antonelli M, Cuenca-Estrella M, Dimopoulos G, Einav S, De Waele J, Garnacho-Montero J, Kanj S, Machado F, Montravers P (2019). ESICM/ESCMID task force on practical management of invasive candidiasis in critically ill patients. Intensive Care Med.

[7] Pappas P, Kauffman C, Andes D, Clancy C, Marr K, Ostrosky-Zeichner L, Reboli A, Schuster M, Vazquez J, Walsh T (2016). Executive summary: clinical practice guideline for the management of candidiasis: 2016 update by the Infectious Diseases Society of America. Clin Infect Dis.

[8] Azoulay E, Dupont H, Tabah A, Lortholary O, Stahl J, Francais A, Martin C, Guidet B, Timsit J (2012). Systemic antifungal therapy in critically ill patients without invasive fungal infection*. Crit Care Med.

[9] Timsit J, Azoulay E, Schwebel C, Charles P, Cornet M, Souweine B, Klouche K, Jaber S, Trouillet J, Bruneel F (2016). Empirical micafungin treatment and survival without invasive fungal infection in adults with ICU-acquired sepsis, Candida colonization, and multiple organ failure: the EMPIRICUS randomized clinical trial. JAMA.

[10] Jaffal K, Poissy J, Rouze A, Preau S, Sendid B, Cornu M, Nseir S (2018). De-escalation of antifungal treatment in critically ill patients with suspected invasive Candida infection: incidence, associated factors, and safety. Ann Intensive Care.

[11] Poissy J, Sendid B, Damiens S, Ishibashi KI, François N, Kauv M, Favory R, Mathieu D, Poulain D (2014). Presence of Candida cell wall derived polysaccharides in the sera of intensive care unit patients: relation with candidaemia and Candida colonisation. Crit Care.

[12] Hanson K, Pfeiffer C, Lease E, Balch A, Zaas A, Perfect J, Alexander B (2012). β-D-glucan surveillance with preemptive anidulafungin for invasive candidiasis in intensive care unit patients: a randomized pilot study. PLoS One.

[13] Nucci M, Nouér S, Esteves P, Guimarães T, Breda G, de Miranda B, Queiroz-Telles F, Colombo A (2016). Discontinuation of empirical antifungal therapy in ICU patients using 1,3-β-d-glucan. J Antimicrob Chemother.

[14] Martín-Mazuelos E, Loza A, Castro C, Macías D, Zakariya I, Saavedra P, Ruiz-Santana S, Marín E, León C (2015). β-D-Glucan and Candida albicans germ tube antibody in ICU patients with invasive candidiasis. Intensive Care Med.

[15] Rautemaa-Richardson R, Rautemaa V, Al-Wathiqi F, Moore C, Craig L, Felton T, Muldoon E (2018). Impact of a diagnostics-driven antifungal stewardship programme in a UK tertiary referral teaching hospital. J Antimicrob Chemother.

[16] Rouzé A, Loridant S, Poissy J, Dervaux B, Sendid B, Cornu M, Nseir S (2017). S-TAFE study group: biomarker-based strategy for early discontinuation of empirical antifungal treatment in critically ill patients: a randomized controlled trial. Intensive Care Med.

[17] Posteraro B, De Pascale G, Tumbarello M, Torelli R, Pennisi M, Bello G, Maviglia R, Fadda G, Sanguinetti M, Antonelli M (2011). Early diagnosis of candidemia in intensive care unit patients with sepsis: a prospective comparison of (1→3)-β-D-glucan assay, Candida score, and colonization index. Crit Care.

[18] Posteraro B, Tumbarello M, De Pascale G, Liberto E, Vallecoccia M, De Carolis E, Di Gravio V, Trecarichi E, Sanguinetti M, Antonelli M (2016). (1,3)-β-d-Glucan-based antifungal treatment in critically ill adults at high risk of candidaemia: an observational study. J Antimicrob Chemother.

[19] Singer M, Deutschman C, Seymour C, Shankar-Hari M, Annane D, Bauer M, Bellomo R, Bernard G, Chiche J, Coopersmith C (2016). The third international consensus definitions for sepsis and septic shock (Sepsis-3). JAMA.

[20] Rhodes A, Evans L, Alhazzani W, Levy M, Antonelli M, Ferrer R, Kumar A, Sevransky J, Sprung C, Nunnally M (2017). Surviving sepsis campaign: international guidelines for management of sepsis and septic shock: 2016. Intensive Care Med.

[21] Colombo A, de Almeida JJ, Slavin M, Chen S, Sorrell T (2017). Candida and invasive mould diseases in non-neutropenic critically ill patients and patients with haematological cancer. Lancet Infect Dis.

[22] Giacobbe D, Mikulska M, Tumbarello M, Furfaro E, Spadaro M, Losito A, Mesini A, De Pascale G, Marchese A, Bruzzone M (2017). Combined use of serum (1,3)-β-D-glucan and procalcitonin for the early differential diagnosis between candidaemia and bacteraemia in intensive care units. Crit Care.

[23] Tissot F, Lamoth F, Hauser P, Orasch C, Flückiger U, Siegemund M, Zimmerli S, Calandra T, Bille J, Eggimann P (2013). β-Glucan antigenemia anticipates diagnosis of blood culture-negative intraabdominal candidiasis. Am J Respir Crit Care Med.

[24] Martínez-Jiménez M, Muñoz P, Valerio M, Alonso R, Martos C, Guinea J, Bouza E (2015). Candida biomarkers in patients with candidaemia and bacteraemia. J Antimicrob Chemother.

[25] Bailly S, Bouadma L, Azoulay E, Orgeas M, Adrie C, Souweine B, Schwebel C, Maubon D, Hamidfar-Roy R, Darmon M (2015). Failure of empirical systemic antifungal therapy in mechanically ventilated critically ill patients. Am J Respir Crit Care Med.

[26] Leroy O, Bailly S, Gangneux J, Mira J, Devos P, Dupont H, Montravers P, Perrigault P, Constantin J, Guillemot D (2016). Systemic antifungal therapy for proven or suspected invasive candidiasis: the AmarCAND 2 study. Ann Intensive Care.

[27] Tabah A, Cotta M, Garnacho-Montero J, Schouten J, Roberts J, Lipman J, Tacey M, Timsit J, Leone M, Zahar J (2016). A systematic review of the definitions, determinants, and clinical outcomes of antimicrobial de-escalation in the intensive care unit. Clin Infect Dis.

[28] Liss B, Cornely O, Hoffmann D, Dimitriou V, Wisplinghoff H (2016). 1,3-ß-D-glucan concentrations in blood products predict false positive post-transfusion results. Mycoses.

[29] Liss B, Cornely O, Hoffmann D, Dimitriou V, Wisplinghoff H (2016). 1,3-β-D-Glucan contamination of common antimicrobials. J Antimicrob Chemother.

[30] Bailly S, Leroy O, Azoulay E, Montravers P, Constantin J, Dupont H, Guillemot D, Lortholary O, Mira J, Perrigault P (2017). Impact of echinocandin on prognosis of proven invasive candidiasis in ICU: a post-hoc causal inference model using the AmarCAND2 study. J Inf Secur.

[31] Urbancic K, Thursky K, Kong D, Johnson P, Slavin M (2018). Antifungal stewardship: developments in the field. Curr Opin Infect Dis.

