# Supplementary material for: (1,3)-β-d-Glucan-based empirical antifungal interruption in suspected invasive candidiasis: a randomized trial
Source: Crit Care. 2020 Sep 5;24:550. doi: 10.1186/s13054-020-03265-y (PMC7487510; doi:10.1186/s13054-020-03265-y)
Supplement: Supplementary file 1 — Additional file 1. Including further details on Methods, Results and six tables (eTable 1, eTable 2, eTable 3, eTable4, eTable 5, eTable 6) and three figures (eFigure 1, eFigure 2, eFigure 3). [file 13054_2020_3265_MOESM1_ESM.docx]

**Electronic Supplement**

**(1,3)-β-D-glucan based antifungal therapy interruption in critically ill septic patients with suspected invasive candidiasis: a randomized trial**

**Methods**

*Definitions*

Proven ICI was defined by: i) histological evidence of yeast cells or hyphae or pseudohyphae from normally sterile site (i.e., fluid obtained by drain during the first 24 hours in place), or ii) positive culture for blood and/or other sterile sites for *Candida* species [1, 2]. Candidaemia was defined as the isolation of *Candida* species in ≥1 blood culture drawn from a patient with consistent clinical manifestations (e.g. fever, chills and/or hypotension). Candidaemia was considered as catheter-related if simultaneous quantitative cultures showed a ratio of ≥5:1 in CFU of blood samples obtained through the catheter and a peripheral vein [3]. A BDG level ≥80 pg/mL was considered as false positive when the patient did not match the above criteria for ICI.

Sepsis and septic shock were classified according to the last international definitions and guidelines (appendix) [4, 5]. The CS and colonization index were calculated when results of the patients’ surveillance cultures (i.e. obtained from urine, skin, or respiratory tract samples) were available. The cut-off points for discriminating between *Candida* species colonization and ICI were ≥3 for the CS and ≥0.5 for the colonization index. The CS for a cut-off value of 3 was as follows: total parenteral nutrition +1, plus surgery + 1, plus multifocal *Candida* colonization + 1, plus sepsis + 2. The colonization index was calculated as the ratio of the number of culture-positive surveillance sites to the total number of sites cultured[6-9]. The following antifungal dosages were considered adequate: fluconazole 800 mg [for obese patients (BMI ≥30), ≥1000 mg) loading dose followed by a daily dosage of at least 400 mg (≥600 for BMI >30 or during CRRT), liposomal amphotericin B (L-AmB) 3 mg/kg/day, amphotericin lipid complex (ABLC) 5 mg/kg/day, caspofungin 70 mg loading dose followed by 50 mg/day (70 mg/day if body weight ≥80 Kg), micafungin 100 mg/day, anidulafungin 200 mg loading dose followed by 100 mg/day[10].

The overall response to antifungal treatment in patients with ICI was assessed in terms of clinical success (resolution of fever, leucocytosis and absence of need for vasopressor support), microbiological success (sterilization of blood and/or negativization of BDG value) and survival. Adequate antifungal therapy was defined as the initiation of antifungal therapy given at a recommended dosage within 48 h after the index blood culture was obtained, with isolation of an organism that was ultimately shown to be susceptible in vitro to the antifungal agent used. Adequate source control was defined as removal of any pre-existing central venous catheters or, if present, other fluid collections thought to be the source of bloodstream infection within 48 h of the incident episode.

*Procedures*

All patients, on the enrolment day, underwent a complete microbiological sampling, including: two sets of blood cultures, respiratory tract sampling (endotracheal aspirate/bronchoalveolar lavage), urine culture, sampling for CCI calculation (as part of microbiological screening), intraoperative sampling (for intra-abdominal and skin/soft tissue infections when surgical source control was performed) or sampling from drainage tubes only if placed less than 24 hours before. Antibiotic therapy was decided by physicians in charge according to local protocols and susceptibility patterns. These included demographic characteristics, medical history, clinical and laboratory findings, the simplified acute physiology score II (SAPS II) [11] and sequential organ failure assessment (SOFA) score (calculated at infection occurrence) [12], duration of antifungal treatment, BDG test results, all microbiological findings including both bacterial and fungal documented infections.

*Microbiological analysis*

Blood samples were obtained for concomitant culture (from a peripheral venepuncture and/or intravascular catheter) and BDG assay (from a peripheral venepuncture and/or an arterial line) following skin and/or catheter disinfection [13]. Blood cultures were processed automatically using a Bactec (BD Diagnostic Systems, Sparks, MD, USA) or BacT/Alert (bioMérieux, Marcy-l’Étoile, France) system, whereas BDG assays were performed by hand on Monday to Friday (from 7:30 a.m. to 7:00 p.m.) and on Saturday (from 7:30 a.m. to 4:00 p.m.). Isolates were identified to the species level by MALDI-TOF MS, supplemented by molecular identification as appropriate [14].Antifungal susceptibility testing was performed as part of routine patient care using the Sensititre Yeast One (Thermo Fisher Scientific, Waltham, MA, USA) colorimetric plate, by which MIC endpoints were visually determined and interpreted according to the current CLSI clinical breakpoints/epidemiological cut-off values to assign susceptibility (or the WT phenotype) to systemic antifungal agents [14].

*Statistical methods*

The Kolmogorov–Smirnov test was used to evaluate the distribution of variables. Data with a non-normal distribution were assessed with the Mann-Whitney test, and the median and selected centile (25th–75th) values are given. The data with a normal distribution were assessed with the Student’s t test. Categorical variables are given as proportions, and were analysed with the chi-square test or Fisher’s exact test, as appropriate. Between-group absolute difference and 95%CI was reported for all outcome variables. *p* < 0.05 was considered significant. All analyses were performed on an intention-to-treat basis. The following parameters of diagnostic performance were calculated for BDG, CS and CCI: sensitivity, specificity, positive and negative predictive value (PPV, NPV), and positive and negative likelihood ratios (+LR, -LR). The discriminatory power for BDG was evaluated by the area under the receiver operating characteristic (ROC) curve (AUC). In the analysis of factors associated with false-positive (1-3)-β-D-glucan results, the crude odds ratio (OR) and 95% CI were calculated for each variable. We included all variables in the multivariable logistic regression if they reached p ≤ 0.2 on univariate analysis. A stepwise selection procedure was used to select variables for inclusion in the final model. The Hosmer–Lemeshow goodness-of-fit test and receiver operating characteristic curve analysis were used to assess the goodness of the logistic final model. Cumulative event curves were estimated with the Kaplan-Meier method.

**Results**

- Median [IQR] duration of ICU and hospital length of stay did not differ between the two groups (18 days [7.75-24.25] vs. 13 days [7-26], p=0.23 and 35 days [23.75-55.25] vs. 38 days [20-59.5], *p*=0.87, respectively). Similarly, no differences were found in the duration of mechanical ventilation and vasopressors use (9 days [4.75-17.25] vs. 9 days [3.25-19.75], *p*=0.97 and 4 days [0.75-8.25] vs. 3 days [0-11], *p*=0.6, respectively). Patients randomized in the two different groups had similar costs due to overall antifungal use (between-group absolute difference in means € 318.63 (-310.1 to 947.3), *p*=0.24), but the expenses attributable to echinocandins use were higher in controls (€ 1320 [618.5-30149.5] vs. € 708 [185.6-1071.5], *p*=0.07). Median cost of the total number of assays in the BDG group was € 80.8±20.4, (table 2).

- Addressing factors associated with false-positive BDG results (continuous renal replacement therapy [CRRT], albumin and immunoglobulin administration, beta-lactam antibiotics treatment, concomitant bacteraemia, use of vacuum-assisted closure devices or surgical gauzes packings), only the concomitant administration of immunoglobulins was independently associated with BG levels >80 pg/mL in absence of ICI (OR 7.43, CI% 95 1.18-46.66, *p*=0.03; AUC±SE 0.76±0.06, 95%CI 0.66-0.84), (eTable 2).

- Diagnostics test indices of BDG, CS and CCI for ICI are shown in Table 1a. BDG showed the best results in all studied variables (100% sensitivity, 79.8% specificity, 100% negative predictive value, 36.7% positive predictive value) with an area under the ROC curve of 0.939, 95% CI (0.875-0.976): 139 pg/mL was the point of the curve with the maximum Younden index, (eTable 3, eTable 4 and eFigure 2).

- Subgroup analysis (surgical vs. non surgical patients) confirmed overall population Primary and secondary outcomes (eTable 5, eTable 6)

- Baseline BDG levels are shown in eFigure 3. The observed relatively high values are due to the inclusion of only the positive results (>80 pg/ml)

**eTable 1. Characteristics of 13 patients with invasive *Candida* infection**

|  | BDG Group  (n=6) | Control Group  (n=7) | *p* value |
| --- | --- | --- | --- |
| ICI at enrolment, N (%) | 6 (100) | 5 (71.4) | 0.51 |
| Subsequent ICI, N (%) | 0 (0.0) | 2 (28.6) | 0.51 |
| *Candida Albicans*, N (%) | 4 (66.7) | 4 (57.1) | 0.41 |
| non*-C. Albicans Candida* species, N (%)* | 2 (33.3) | 3 (42.9) | 0.4 |
| Candidemia, N (%) | 4 (66.7) | 6 (85.7) | 0.88 |
| - Abdominal source, N (%) | 0 (0.0) | 1 (14.3) | 0.93 |
| - Urinary source, N (%) | 2 (33.3) | 1 (14.3) | 0.88 |
| - CVC-associated infection, N (%) | 0 (0.0) | 2 (28.6) | 0.51 |
| - Unknown source, N (%) | 2 (33.3) | 2 (28.6) | 0.67 |
| Abdominal candidiasis, N (%) | 2 (33.3) | 1 (14.3) | 0.88 |
| SOFA score, median [IQR] | 12 [7-14.0] | 10 [5-11.75] | 0.48 |
| Septic shock, N (%) | 5 (83.3) | 6 (85.7) | 0.52 |
| Duration of antifungal therapy, mean days [IQR] | 15 [9-26.0] | 17 [12.25-23.25] | 0.89 |
| Initial BG values, pg/mL** | 598.5 [200-880] | 258 [242.25-500] | 0.58 |
| Clinical cure, N (%) | 3 (50.0) | 5 (71.4) | 0.83 |
| Microbiological eradication, N (%) | 4 (66.7) | 4 (57.1) | 0.83 |
| Adequate antifungal therapy, N (%) | 6 (100) | 7 (100) | - |
| Adequate source control, N (% )*** | 2 (33.3) | 4 (57.1) | 0.76 |
| 30-day mortality, N (%) | 3 (50.0) | 3 (42.9) | 0.77 |

ICI: invasive *Candida* infection; SOFA: Sequential Organ Failure Assessment; CVC: central venous catheter; BG: (1-3)-β-D-glucan

*The following species were identified: *Candida Parapsilosis* (n=2), *Candida Tropicalis* (n=1), *Candida Glabrata* (n=1), *Candida Dubliniensis* (n=1)

**Initial BG values were available in 11 patients with ICI at enrollment.

*** In four cases the source was unknown. In one case of abdominal source and cenral-line associated infection, the sources were not promptly adequately controlled.

**eTable 2. Univariate and multivariate analyses of factors associated with false-positive (1-3)-β-D-glucan results ***

|  | **No. (%) of patients** | | **Crude analysis** | | **Adjusted analysis** | |
| --- | --- | --- | --- | --- | --- | --- |
| Variable | False Positive  (*n*=19) | True Negative  (*n*=75) | *p* value | OR  (95% CI) | *p* value | OR  (95% CI) |
| Medical admission, N(%) | 10 (52.6) | 38 (50.7) | 0.88 | 1.08  (0.39-2.96) | .. | .. |
| Surgical admission, N(%) | 7 (36.8) | 27 (36) | 0.95 | 1.04 (0.36-2.95) | .. | .. |
| Trauma admission, N(%) | 2 (10.5) | 10 (13.3) | 0.74 | 0.77 (0.15-3.82) | .. | .. |
| SAPS II score | 44  [28-56.5] | 40  [32.3-52] | 0.91 | 1.001  (0.97-1.03) | .. | .. |
| Charlson score | 3  [2-4.75] | 4  [1-6.75] | 0.47 | 0.95 (0.81-1.1) | .. | .. |
| SOFA score | 6 [4-11] | 7 [4-10] | 0.9 | 1.01 (0.88-1.16) | .. | .. |
| Septic shock, N(%) | 13 (68.4) | 30 (40) | 0.03 | 3.25 (1.11-9.5) | 0.08 | 2.9 (0.88-9.61) |
| Hospital LOS before randomization, (days) | 8 [3.25-13.75] | 8 [4-13] | 0.39 | 1.01 (0.98-1.04) | .. | .. |
| ICU LOS before randomization, (days) | 3 [2-8] | 3 [2-7.5] | 0.77 | 1.01 (0.93-1.1) | .. | .. |
| Multifocal *Candida* colonization, N (%) | 6 (31.6) | 24 (32) | 0.97 | 0.98 (0.33-2.89) | .. | .. |
| Abdominal surgery, N (%) | 13 (68.4) | 50 (66.7) | 0.88 | 1.08 (0.37-3.19) | .. | .. |
| AKI requiring CRRT, N (%) | 5 (2.6) | 10 (13.3) | 0.18 | 2.32 (0.69-7.86) | 0.56 | 1.45 (0.4-5.56) |
| Albumin administration, N (%) | 15 (78.9) | 55 (73.3) | 0.61 | 1.36 (0.4-4.6) | .. | .. |
| Immunoglobulins administration, N (%) | 3 (15.8) | 4 (5.3) | 0.14 | 3.33 (0.68-16.36) | **0.03** | **7.43 (1.18-46.66)** |
| Beta-lactam antibiotics administration, N (%) | 18 (94.7) | 61 (81.3) | 0.18 | 4.13 (0.51-33.6) | 0.25 | 3.7 (0.4-34) |
| Bacterial bloodstream infection, N(%) | 5 (26.3) | 12 (16) | 0.3 | 1.86 (0.57-6.18) | .. | .. |
| VAC device/packing with surgical gauzes, N(%) | 6 (31.6) | 10 (13.3) | 0.07 | 3 (0.93-9.71) | 0.07 | 3.28 (0.92-11.7) |

Data are presented as median (IQR), unless otherwise indicated

SAPS II: Simplified Acute Physiology Score; SOFA: Sequential Organ Failure Assessment; LOS: length of stay; ICU: Intensive Care Unit; AKI: acute kidney injury; CRRT: continuous renal replacement therapy; IQR: interquartile range; VAC: vacuum-assisted closure

*The analyses included 94 patients (11 patients with ICI at inclusion and three patients in the control group without baseline BG value were excluded).

**eTable 3. Performances of (1-3)-β-D-glucan (≥80 pg/mL), *Candida* score and *Candida* colonization index for ICI diagnosis**

| Invasive candidiasis | Sensitivity | Specificity | PPV | NPV | +LR | -LR |
| --- | --- | --- | --- | --- | --- | --- |
| BG ≥80 pg/mL (%)* | 100 | 79.8 | 36.7 | 100 | 4.95 | 0 |
| CS ≥ 3 (%) | 81.8 | 52.6 | 16.4 | 96.2 | 1.73 | 0.35 |
| CCI ≥0.5 (%) | 63.6 | 71.1 | 20 | 94.5 | 2.2 | 0.51 |

BG: (1-3)-β-D-glucan; CS: *Candida* score; CCI: *Candida* colonization index; ICI: invasive *Candida* infection; PPV: positive predictive value; NPV: negative predictive value; +LR: positive likelihood ratio; -LR: negative likelihood ratio

*Initial BG values were available in 105 patients

**eTable 4. Cut-off values for the ROC curve**

| BG cut-off value  (pg/mL) | Sensitivity | 95% CI | Specificity | 95% CI | +LR | -LR |
| --- | --- | --- | --- | --- | --- | --- |
| >139 | **100** | **71.5-100** | **85.11** | **76.3-91.6** | **6.71** | **0** |
| >141 | 90.91 | 58.7-99.8 | 85.11 | 76.3-91.6 | 6.1 | 0.11 |
| >190 | 90.91 | 58.7-99.8 | 89.36 | 81.3-94.8 | 8.55 | 0.1 |
| >200 | 81.82 | 48.2-97.7 | 89.36 | 81.3-94.8 | 7.69 | 0.2 |
| >212 | 81.82 | 48.2-97.7 | 90.43 | 82.6-95.5 | 8.55 | 0.2 |
| >258 | 54.55 | 23.4-83.3 | 90.43 | 82.6-95.5 | 5.7 | 0.5 |
| >286 | 54.55 | 23.4-83.3 | 92.55 | 85.3-97 | 7.32 | 0.49 |
| >437 | 45.45 | 16.7-76.6 | 92.55 | 85.3-97 | 6.1 | 0.59 |
| >466 | 45.45 | 16.7-76.6 | 95.74 | 89.5-98.8 | 10.68 | 0.57 |
| >500 | 27.27 | 6-61 | 98.94 | 94.2-100 | 25.64 | 0.74 |

ROC: receiver operating characteristics; BG: (1-3)-β-D-glucan

**eTable 5. Outcome measures in the (1-3)-β-D-glucan (BDG) and the control groups (non-surgical patients)**

| Variable | BDG Group  (n=39) | Control Group  (n=30) | Between-group absolute  difference in means  (95% CI) | *p* value |
| --- | --- | --- | --- | --- |
| **Primary outcome** | | | | |
| Duration of antifungal therapy, days | 2 [1-2.75] | 9 [6-13] | 6.69 (3.92 to 9.47) | **<0.001** |
| **Secondary outcomes** | | | | |
| 30 day mortality, N (%) | 11 (28.2) | 11 (36.7) | 8.5% (-15.13 to 32.03) | 0.62 |
| ICU mortality, N (%) | 13 (33.3) | 12 (40) | 6.7% (-17.5 to 30.76) | 0.75 |
| Hospital mortality, N %) | 14 (35.9) | 13 (43.3) | 7.4% (-17.2 to 31.6) | 0.71 |
| Subsequent ICI, N (%)* | 0 | 2 (6.7) | 6.7% (-4.07 to 22.12) | 0.36 |
| Hospital LOS, days | 36 [24.25-54.25] | 40 [24-66] | -1.98 (-21.47 to 17.5) | 0.84 |
| ICU LOS, days | 19 [11-25.75] | 21.5 [7-38] | 5.03 (-4.07 to 14.12) | 0.27 |
| Mechanical ventilation duration, days | 10 [5.25-18.75] | 16.5 [5-27] | 8.57 (1.2 to 15.94) | **0.02** |
| Vasopressors duration, days | 5 [1.25-13.25] | 4.5 [0-14] | 1.42 (-2.82 to 5.65) | 0.51 |
| Total antifungals costs, € | 110 [2.64 -708] | 218 [11-1242.5] | 580.3 (-274.7 to 1435.3) | 0.18 |

Data are presented as median (IQR) and N (%). Between-group absolute differences are calculated using the mean values, percentage differences, and 95% CIs.

BDG: (1-3)-β-D-glucan; ICU: Intensive Care Unit; ICI: invasive *Candida* infection; LOS: length of stay; ICU: Intensive Care Unit; €: Euro; IQR: interquartile range

*See eTable 1 for further details

**eTable 6. Outcome measures in the (1-3)-β-D-glucan (BDG) and the control groups (surgical patients)**

| Variable | BDG Group  (n=14) | Control Group  (n=25) | Between-group absolute  difference in means  (95% CI) | *p* value |
| --- | --- | --- | --- | --- |
| **Primary outcome** | | | | |
| Duration of antifungal therapy, days | 2 [1-3] | 10 [6-13.5] | 5.11 (0.4 to 9.85) | **0.001** |
| **Secondary outcomes** | | | | |
| 30 day mortality, N (%) | 4 (28.6) | 4 (16) | 12.6% (-15.88 to 44.28) | 0.62 |
| ICU mortality, N (%) | 3 (21.4) | 5 (20) | 1.4% (-25.23 to 33.58) | 0.76 |
| Hospital mortality, N %) | 5 (35.7) | 5 (20) | 15.7% (-15.2 to 47.7) | 0.49 |
| Subsequent ICI, N (%)* | 0 | 0 | - | - |
| Hospital LOS, days | 22.5 [15-36] | 25 [11.75-37.75] | -12.08 (-32.6 to 8.5) | 0.24 |
| ICU LOS, days | 12.5 [7-22] | 8 [6.5-13.25] | -4.83 (-11.1 to 1.46) | 0.13 |
| Mechanical ventilation duration, days | 5 [1-14] | 4 [2.75-11-27] | -1.79 (-6.6 to 3.04) | 0.46 |
| Vasopressors duration, days | 1 [0-3] | 1 [0-3] | -1.77 (-5.29 to 1.74) | 0.31 |
| Total antifungals costs, € | 342 [2.02 -1062] | 15.84 [8.36-1182.5] | -105.165 (-1033.2 to 822.8) | 0.82 |

Data are presented as median (IQR) and N (%). Between-group absolute differences are calculated using the mean values, percentage differences, and 95% CIs.

BDG: (1-3)-β-D-glucan; ICU: Intensive Care Unit; ICI: invasive *Candida* infection; LOS: length of stay; ICU: Intensive Care Unit; €: Euro; IQR: interquartile range

*See eTable 1 for further details

**eFigure 1. Study protocol**


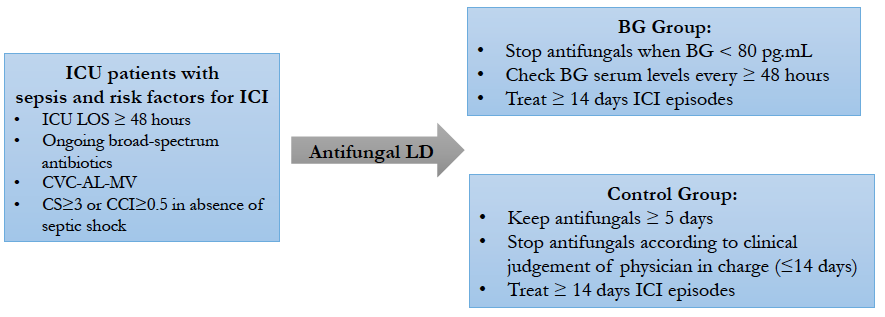


**Legend**

ICU: Intensive Care Unit; CVC: central venous catheter; AL: arterial line; MV: mechanical ventilation; CD: *Candida* Score; CCI: *Candida* Colonization Index; LD: loading dose; BG: (1-3)-β-D-Glucan; ICI: Invasive *Candida* Infection

**eFigure 2. ROC AUC curve for ICI diagnosis**

The area under the ROC curve was 0.939, 95%CI (0.875-0.976) . The point on the curve with the maximum Youden index was 139 pg/mL. Diagnostic performance of the marker is detailed in Table 1a

**Legend**

ROC: receiver operating characteristics; AUC: area under the curve, ICI: invasive *Candida* infection

**eFigure 3. Initial detectable BDG serum levels**

**
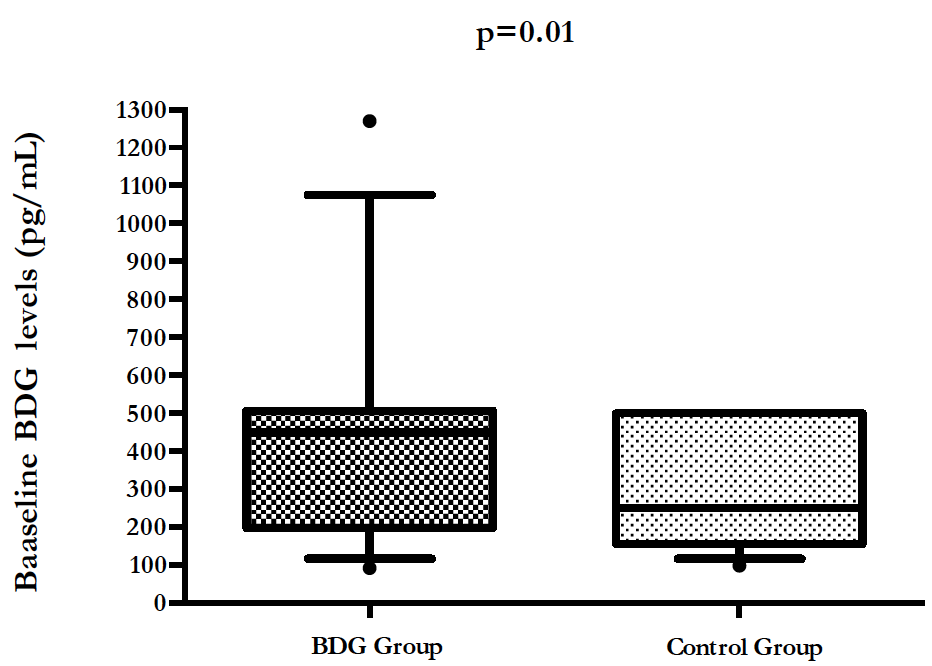
**

**References**

1. Martin-Loeches I, Antonelli M, Cuenca-Estrella M, Dimopoulos G, Einav S, De Waele J, Garnacho-Montero J, Kanj S, Machado F, Montravers P *et al*: **ESICM/ESCMID task force on practical management of invasive candidiasis in critically ill patients**. *Intensive Care Med* 2019, **45**(6):789-805.

2. Pappas P, Kauffman C, Andes D, Clancy C, Marr K, Ostrosky-Zeichner L, Reboli A, Schuster M, Vazquez J, Walsh T *et al*: **Executive Summary: Clinical Practice Guideline for the Management of Candidiasis: 2016 Update by the Infectious Diseases Society of America**. *Clin Infect Dis* 2016, **62**(4):409-417.

3. Posteraro B, Tumbarello M, De Pascale G, Liberto E, Vallecoccia M, De Carolis E, Di Gravio V, Trecarichi E, Sanguinetti M, Antonelli M: **(1,3)-β-d-Glucan-based antifungal treatment in critically ill adults at high risk of candidaemia: an observational study.** *J Antimicrob Chemother* 2016, **71**(8):2262-2269.

4. Singer M, Deutschman C, Seymour C, Shankar-Hari M, Annane D, Bauer M, Bellomo R, Bernard G, Chiche J, Coopersmith C *et al*: **The Third International Consensus Definitions for Sepsis and Septic Shock (Sepsis-3)**. *JAMA* 2016, **315**(8):801-810.

5. Rhodes A, Evans L, Alhazzani W, Levy M, Antonelli M, Ferrer R, Kumar A, Sevransky J, Sprung C, Nunnally M *et al*: **Surviving Sepsis Campaign: International Guidelines for Management of Sepsis and Septic Shock: 2016**. *Intensive Care Med* 2017, **43**(3):304-377.

6. León C, Ruiz-Santana S, Saavedra P, Almirante B, Nolla-Salas J, Alvarez-Lerma F, Garnacho-Montero J, León M, EPCAN Study Group: **A bedside scoring system ("Candida score") for early antifungal treatment in nonneutropenic critically ill patients with Candida colonization**. *Crit Care Med* 2006, **34**(3):730-737.

7. León C, Ruiz-Santana S, Saavedra P, Galván B, Blanco A, Castro C, Balasini C, Utande-Vázquez A, Molina FGd, Blasco-Navalproto M *et al*: **Usefulness of the "Candida score" for discriminating between Candida colonization and invasive candidiasis in non-neutropenic critically ill patients: a prospective multicenter study**. *Crit Care Med* 2009, **37**(5):1624-1633.

8. Eggimann P, Pittet D: **Candida colonization index and subsequent infection in critically ill surgical patients: 20 years later**. *Intensive Care Med* 2014, **40**(10):1429-1448.

9. Pittet D, Monod M, Suter P, Frenk E, Auckenthaler R: **Candida colonization and subsequent infections in critically ill surgical patients.** *Ann Surg* 1994, **220**(6):751-758.

10. Bassetti M, Righi E, Ansaldi F, Merelli M, Trucchi C, De Pascale G, Diaz-Martin A, Luzzati R, Rosin C, Lagunes L *et al*: **A multicenter study of septic shock due to candidemia: outcomes and predictors of mortality**. *Intensive Care Med* 2014, **40**(6):839-845.

11. Le Gall J, Lemeshow S, Saulnier F: **A new Simplified Acute Physiology Score (SAPS II) based on a European/North American multicenter study**. *JAMA* 1993, **270**(24):2957–2963.

12. Ferreira F, Bota D, Bross A, Mélot C, Vincent J: **Serial evaluation of the SOFA score to predict outcome in critically ill patients**. *JAMA* 2001; , **286**(14):1754–1758.

13. Posteraro B, De Pascale G, Tumbarello M, Torelli R, Pennisi M, Bello G, Maviglia R, Fadda G, Sanguinetti M, Antonelli M: **Early diagnosis of candidemia in intensive care unit patients with sepsis: a prospective comparison of (1→3)-β-D-glucan assay, Candida score, and colonization index**. *Crit Care* 2011, **15**(5):R249.

14. Posteraro B, Spanu T, Fiori B, De Maio F, De Carolis E, Giaquinto A, Prete V, De Angelis G, Torelli R, D'Inzeo T *et al*: **Antifungal susceptibility profiles of bloodstream yeast isolates by Sensititre YeastOne over nine years at a large Italian teaching hospital**. *Antimicrob Agents Chemother* 2015, **59**(7):3944-3955.
